# Evaluation of Organosolv Lignin as an Oxidation Inhibitor in Bitumen

**DOI:** 10.3390/molecules25102455

**Published:** 2020-05-25

**Authors:** Yi Zhang, Xueyan Liu, Panos Apostolidis, Ruxin Jing, Sandra Erkens, Natascha Poeran, Athanasios Skarpas

**Affiliations:** 1 School of Highway, Chang’an University, Xi’an 710064, China; yizhang@chd.edu.cn; 2 Faculty of Civil Engineering and Geosciences, Delft University of Technology, Stevinweg 1, 2628 CN Delft, The Netherlands; P.Apostolidis@tudelft.nl (P.A.); R.Jing@tudelft.nl (R.J.); s.m.j.g.erkens@tudelft.nl (S.E.); athanasios.skarpas@ku.ac.ae (A.S.); 3 Boskalis Nederland B.V., 2150 AD Nieuw-Vennep, The Netherlands; natascha.poeran@boskalis.com; 4 Department of Civil Infrastructure and Environmental Engineering, Khalifa University of Science and Technology, Abu Dhabi 127788, UAE

**Keywords:** organosolv lignin, oxidation inhibitor, bitumen, aging, microstructure, chemistry, rheology

## Abstract

Organosolv lignin, a natural polymer, has been used in this study as an oxidation inhibitor in bitumen. Particularly, the effect of oxidative aging on the chemical compositional changes and on the rheology of bituminous binders with organosolv lignin and the impact to inhibit oxidation in bitumen were evaluated. Firstly, after analyzing the microstructure and surface characteristics of utilized organosolv lignin, a high shear mixing procedure was followed to produce binders of different proportions of lignin in bitumen. Pressure aging vessel conditioning was applied to these binders to simulate in-field aging and a series of tests were performed. Fourier transform infrared spectroscopy was used to track the compositional changes of lignin–bitumen systems before and after aging respectively. The rheological changes due to oxidative aging in the different lignin–bitumen systems were studied by means of dynamic shear rheometer tests. Based on the spectroscopic laboratory analyses, certain proportions of organosolv lignin in bitumen have shown a potential oxidation retardation effect in bitumen since a reduction of carbonyl and sulfoxide compounds was observed. However, the addition of lignin reduced the fatigue life of bitumen and potentially led to an increase in brittle fracture sensitivity at low and medium temperatures. Nevertheless, lignin improved the rutting resistance at high temperatures. Overall, it can be concluded that organosolv lignin can suppress the oxidation of sulfur and carbon compounds in bitumen either by direct deceleration of oxidation reaction or interaction with compounds that otherwise are oxidizable, without seriously degrading the mechanical properties.

## 1. Introduction

Bitumen is a complex petroleum-based material that is the most widely used binder for paving applications. However, considering the uncertainty in crude oil supply, alternative binders is encouraged to be used as a replacement of bituminous binders or performance modifiers. Especially, lignin, among others, has attracted considerable attention as modifier [1,2] or substitute [3] of bitumen. Lignin is one of the most abundant natural polymers on Earth, with the total amount of lignin present in the biosphere estimated to exceed 300 billion tons and with an annual increase of approximately 20 billion tons [4]. Lignin can be found as well in coproducts of timber production, or byproducts of paper and pulp industries. Thus, the utilization of lignin in binders specially designed for pavements may bring large economic benefits to sustainable development.

The use of lignin is not limited only as modifier or substitute of bitumen but it can be incorporated as an oxidation inhibitor as well to minimize the aging potential and to improve the durability of binders [5]. Particularly, during oxidative aging, oxygen reacts with molecules present in bitumen, producing polar compounds, principally ketones and sulfoxides and causing an increase in the portion of asphaltenes. In general, oxidation reactions in bitumen yield changes in its generic chemical composition and, finally, its colloidal structure, deteriorating the physicomechanical properties. Lignin has been found to retard bitumen oxidation [6,7,8,9], mainly due to the lignin radical scavenging activity, its polyphenolic structure [10,11,12] and the physical interaction between bitumen and lignin [1]. However, lignin itself also has a large number of carbonyl and sulfoxide functional groups, which should be taken into account separately from the bituminous functional groups that evolve due to the aging of bitumen [13]. Different types of lignin can be obtained through different extraction methods. The main extraction methods include the soda, kraft, sulfite and organosolv processes and steam explosion [14,15,16].

Within this framework, a laboratory study is performed to elucidate the effect of lignin on bitumen in terms of oxidative aging by performing a series of tests. The studied binders, collectively named lignin–bitumen systems, were oxidized by the standard pressure-aging vessel (PAV) procedure, the oxidation induced compositional changes, and the alterations in glass transition temperature and rheology were investigated by comprehensive analyses. Overall, this study was designed to examine the effect of organosolv lignin, of various proportions, on the oxidation of bitumen.

## 2. Materials and Methods

### 2.1. Characteristics of Organosolv Lignin

**Figure 1:**
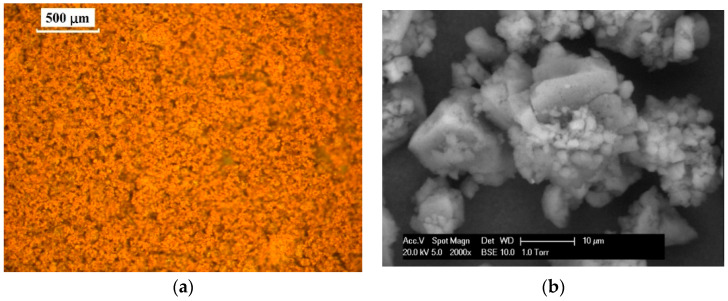
Microscope images of organosolv lignin powder; (**a**) optical microscope and (**b**) environmental scanning electron microscope (ESEM) image of lignin powder.

As mentioned earlier, the organosolv lignin powder was selected in this study to be used as an oxidation inhibitor and potentially as a bitumen modifier. This type of lignin (brownish powder) was provided by Chemical Point UG, Oberhaching, Germany. It had a purity of above 87% and was used without any extra purification. The size of lignin powder, which was in the range of 20–200 μm, observed by an optical microscope and an environmental scanning electron microscope (ESEM, Eindhoven, Netherlands). The procedure to prepare the ESEM device and samples is discussed in [1]. The lignin powder was fixed on the specific sample holder plate with a black sticker sheet. The optical microscopic and ESEM images of lignin before its addition in bitumen are demonstrated in Figure 1a,b, respectively. As shown in Figure 1b, the surface of lignin particles was rough and of high angularity.

Additionally, the specific surface area of lignin was calculated by the Braunauer–Emmett–Teller (BET) method after a measurement conducted by a dynamic vapor sorption (DVS, London, UK) surface measuring device. During the measurements in DVS, 30 mg of organsolv lignin was used at 25 °C under 100 sccm flow rate and 0.1% (P/P0) partial pressure. For the BET calculations, a monolayer coverage is assumed as the only physical adsorption mechanism and 147.0593 m^2^/g specific surface area of lignin was obtained. Also, the density of lignin was measured as 1.3774 g/cm^3^ by a helium pycnometer.

### 2.2. Preparation of Lignin–Bitumen Systems

Bitumen blended with a significant amount of organosolv lignin (25% w/w of the mix) has shown quite improved performance characteristics [3]. In this study, various contents of lignin were added in a 70/100 pen grade bitumen with a softening point of 47.5 °C. An overview of the studied materials is provided in Table 1.

The mixing time and temperature were determined by comparing different combinations and referred to the previous experimental experience. The high shear mixer was applied in the process, the mixing temperature was 163 °C and the shearing speed was 3000 rpm. The lignin was added in the bitumen gradually. When it was all added, the stirring lasted for 30 min until the mix became homogenous and bubbles were eliminated.

The aging process was simulated by the PAV, which was implemented after the short-term aging procedure in accordance with ASTM D 6521-19 [17]. A 3.2 mm-thick film of bitumen was formed after pouring 50 ± 0.5 g of virgin bitumen into the standard PAV pan. The PAV device was set at an air pressure of 2.10 MPa at 100 °C. The whole aging process lasted 20 h.

### 2.3. Fourier Transform Infrared Spectroscopic Measurements

In this study, a Fourier transform infrared (FTIR, Waltham, MA, USA) spectrometer (100 FTIR Perkin Elmer) was used with a single-point attenuated total reflectance (ATR) fixture to collect the spectra data of lignin and bitumen samples. The wavenumber ranged from 600 to 4000 cm^−1^ with a resolution of 4 cm^−1^. Nine replications for each sample were analyzed. Different functional groups have a different light-absorption spectrum. The functional group absorbance index (*AI*) was used for the main absorption bands of lignin to compare the changes of functional groups with the changes of spectra and it was determined as

(1) $$AI=\frac{A_{ab}}{\sum A}$$

where *A_ab_* is the integral area of absorption band ab, ∑*A* is the sum of the integral areas of several characteristic functional group peaks. The wavenumbers of typical bands of chemical functional groups to be calculated and considered are summarized in Table 2.

The carbonyl (C=O, 1700 cm^−1^) and sulfoxide (S=O, 1030 cm^−1^) compounds were analyzed as conventional aging indices for both neat bitumen and lignin–bitumen systems. The aging effect of lignin itself was not obvious or even negligible [1]. To eliminate the effect of lignin, as a material of high content of carbonyls and sulfoxides, the increase ratio index (*IRI*) was calculated by using the difference of the aging index under fresh and aged conditions, and the proportion of bitumen in the mix. The *IRI* defined the rate of growth of aging chemical functional groups per unit mass of bitumen from the fresh to the aged state. The equation is as follows

(2) $$IRI=\frac{AI_{Aged}-AI_{Fresh}}{W}$$

where *AI*_Aged_ is the aging index of samples in aged condition; *AI*_Fresh_ is the aging index of samples in unaged condition; *W* is the mass fraction of bitumen in the mix (*m_bit_*/*m_total_*). The higher the *IRI* value is, the faster the aging functional groups increase at the same aging process and vice versa.

### 2.4. Rheological Measurements

#### 2.4.1. Frequency Sweep

To characterize the rheological properties, complex shear modulus (G*) and phase angle (*δ*), of bitumen over a wide range of temperatures and frequencies were performed by the dynamic shear rheometer (DSR) Anton Paar MCR 502 under temperature-frequency sweeps according to AASHTO T 315-19 [18] on unaged and PAV-aged samples. In this study, the parallel-plates geometry with an 8 mm diameter and 2 mm gap was used at the range of temperature from −10 to 30 °C with 10 °C increments. For the relatively elevated temperature range of 30 to 60 °C with 10 °C increments 25 mm-diameter plates and 1 mm-gap were used. The samples were tested with a frequency sweep from 100 to 0.1 rad/s at each temperature. The master curves of the G* and *δ* were constructed by the results from temperature-frequency sweeps at a reference temperature of 20 °C. The effect of the different contents of lignin and aged conditions were depicted by the results of master curves. The sigmoidal model was applied to fit the results to obtain a single smooth master curve. The sigmoidal function model equation is given as follows

(3) log|G*|=δ+α1+1e(β+γlogfR)

where *f_R_* is reduced frequency; *δ* is the minimum value of |G*|; *α* is the difference between the maximum and minimum value of |G*|; and *β, γ* are the fitting coefficients of the sigmoidal model.

#### 2.4.2. Linear Amplitude Sweep

The fatigue behavior of neat bitumen and lignin–bitumen binders were assessed by the linear amplitude sweep (LAS) test with cyclic loading. The strain amplitude was increasing linearly in accordance with AASHTO TP 101-14 [19]. The geometry of the measurement device used in the LAS test was the 8 mm-diameter parallel plates with a 2 mm gap. The LAS test included two stages. Firstly, the frequency sweep test was used to determine the rheological performance of the bitumen. The frequency sweep was tested at 20 °C and subjected to oscillatory shear loading at constant amplitude over a range of loading frequencies, with an applied load of 0.1% strain over a range of frequencies from 0.2~30 Hz, whereby data were sampled at the following 12 typical frequencies: 0.2, 0.4, 0.6, 0.8, 1.0, 2.0, 4.0, 6.0, 8.0, 10, 20, and 30 Hz. Then, in the second step, the samples were subjected to a strain sweep the frequency of which was 10 Hz. The LAS test was performed at a temperature of 20 °C to evaluate the fatigue properties of the studied samples. Oscillatory strain-controlled cycles with linearly increasing strain amplitudes from 0 to 30% were conducted to accelerate the fatigue damage of bitumen.

#### 2.4.3. Multiple Stress Creep Recovery

The presence of elastic response in bitumen and the change in elastic response at two different stress levels were identified by the multiple stress creep recovery (MSCR) method in accordance with ASTM D 7405-15 [20]. Nonrecoverable creep compliance was an indicator of the resistance of bitumen to permanent deformation with a repeated load. The properties could be better indicated by the higher stress levels applied in MSCR and generated rearrangement and fracture of the polymer network. Two stress levels, 100 Pa and 3200 Pa, were performed in this test. The samples were subjected to a creep load for 1 s and recovered under zero stress for 9 s after the removal of the load in every cycle. First, 20 cycles were applied for the lower stress level (0.1 kPa) and an additional 10 cycles under the higher stress level (3.2 kPa) after that. The first 10 cycles at 0.1 kPa were designed for the conditioning of samples. The temperature range was from 52 to 70 °C and the increment was 6 °C (52, 58, 64, and 70 °C). The percent recovery indicates the tendency to recover to the original state of bitumen. The nonrecoverable creep compliance describes the nonrecoverable strain after the load is removed. These two important indicators evaluate the resistance to permanent deformation which is typical pavement distress at high temperature.

## 3. Results and Discussion

### 3.1. Compositional and Glass Transition Changes

**Figure 2:**
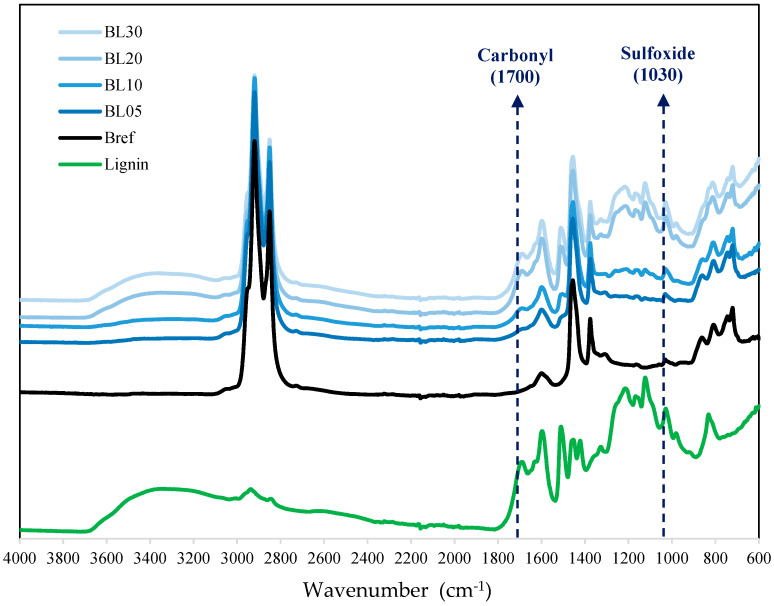
Fourier transform infrared spectroscopy (FTIR) spectra of lignin–bitumen systems.

The FTIR spectra and the compositional changes of different content lignin–bitumen systems were plotted in Figure 2. The functional group peaks of these systems can be found in both base materials (bitumen and lignin), and these peaks corresponded one to one. It shows that mixing lignin powder and virgin bitumen did not lead to the production of new chemical species, because no new chemical functional group peaks were generated.

**Figure 3:**
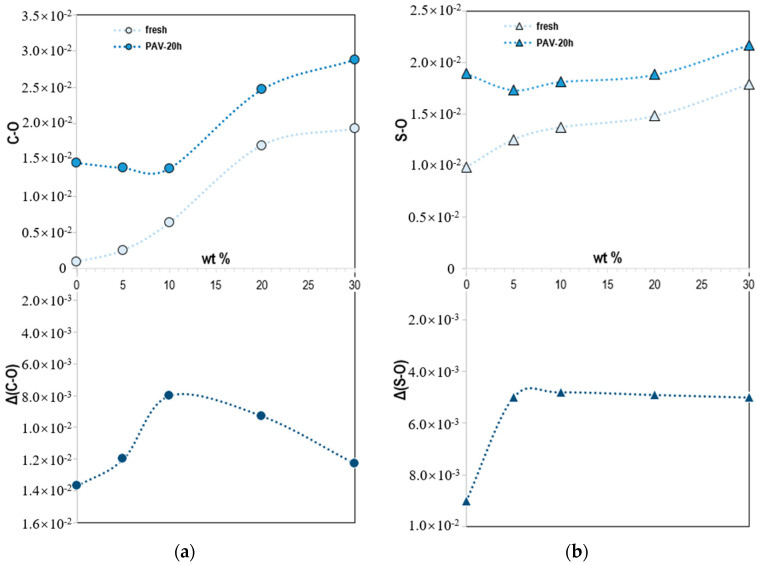
The (**a**) carbonyl (1700 cm^−1^) and (**b**) sulfoxide (1030 cm^−1^) indices of lignin–bitumen systems and their incremental values before and after pressure-aging vessel (PAV) aging.

The values of aging indices were the average of nine measurements and summarized in Figure 3. Obviously, these values were higher than those of fresh samples. As the aging progressed, the components inside the bitumen reacted to form more carbonyl and sulfoxide compounds. In comparison to the fresh samples, the aging indices increased with the increase of lignin content. The order of the values strictly follows the order of lignin content. The greater the lignin content, the greater the value. It shows that lignin itself contains these two functional groups and the carbonyl compounds grew more noticeably than sulfoxide ones with the increase of lignin dosage.

By comparing fresh and aged samples, the addition of lignin significantly slowed down the generation of aged functional groups of bitumen. It shows that the lignin has a certain oxidation inhibition effect on the bitumen. Particularly, the use of *IRI* helped to improve the interpretation of the oxidation inhibition effect of lignin in bitumen. The higher the increase ratio index (*IRI*) value is, the faster the aging functional groups increase at the same aging time and vice versa. Apparently, the addition of lignin declined the growth rate of both carbonyl and sulfoxide compounds in comparison to bitumen. However, the changes in the two functional groups with the content of lignin were different. The increase rate of the carbonyl group decreased first and then increased as the content of lignin increased. The *IRI* reached its lowest level when the content of lignin was 10%. However, the increased rate of sulfoxide basically did not change with the lignin content. The *IRI* of sulfoxide was slightly different from each other compared with that of the carbonyl compound but was significantly lower than of neat bitumen.

From the chemistry point of view, the top surface of bitumen, which is exposed to the external environment and thus oxygen, ages first. As time proceeds, bitumen starts to be oxidized internally due to the diffusion of oxygen. In the case of bitumen with organosolv lignin particles, the diffusion of oxygen is potentially prohibited or the oxygen could not find an adequate volume of sensitive carbon and sulfur molecules of bitumen to react with and to produce carbonyl and sulfoxide compounds. In other words, it would take a longer time for the oxygen to diffuse and react with these bituminous species. Based on the spectroscopic results, no new reaction products were observed with the addition of lignin in bitumen and thus it is believed that lignin possibly can deactivate oxidation promotors and oxygen receptors that might be in bitumen. The mechanism of inhibition of oxidation is thus based on prohibiting the susceptible to oxygen species to transform to carbonyl or sulfoxide compounds decelerating thus the overall oxidation process of bitumen.

### 3.2. Rheological Changes

#### 3.2.1. Viscoelastic Properties

Complex modulus and phase angle at various temperature ranges (−10 to 60 °C) were obtained by performing frequency sweep tests. The data in each temperature range were fitted by the sigmoidal function model (see Equation (3)). Finally, the master curves with reduced frequency as the *x*-coordinate axis, complex modulus, and phase angle as the *y*-coordinate axis were formed. A wide range of frequencies was addressed. A higher modulus of binder implied a higher resistance to the deformation. While a lower phase angle indicated a more elastic binder.

**Figure 4:**
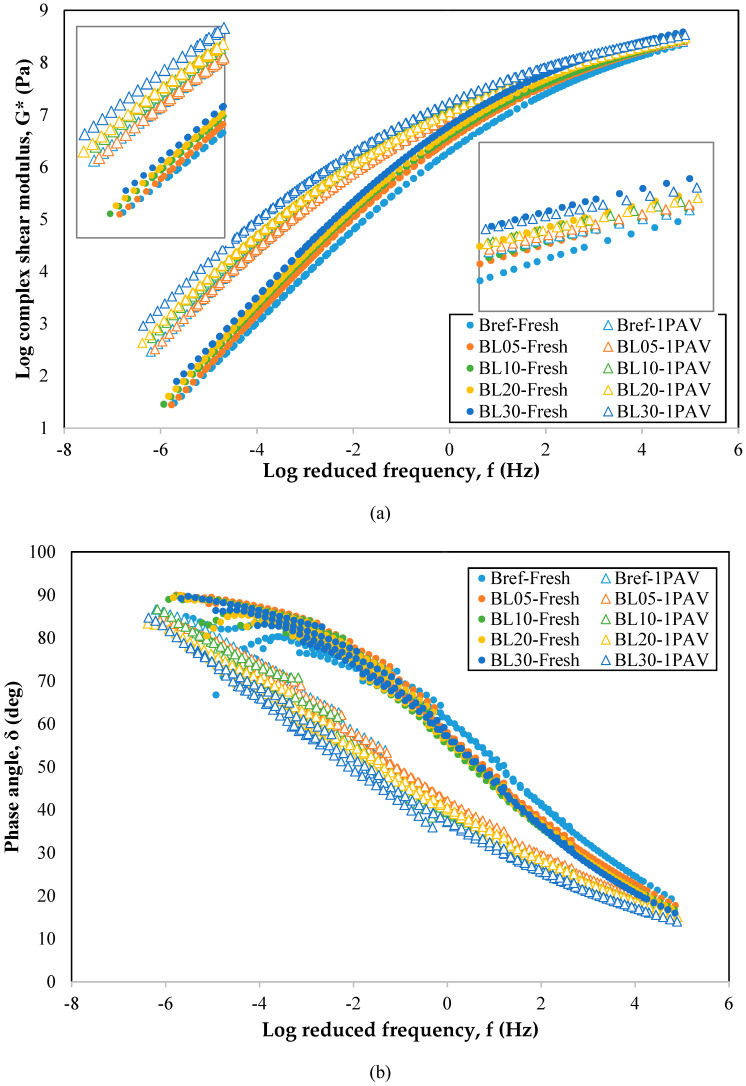
Master curves of (**a**) complex shear modulus and (**b**) phase angle.

Each binder had been tested with three samples and the master curves of complex modulus and phase angle of different binders are shown in Figure 4a,b, respectively. A slight increase of modulus and decrease of phase angle were observed with the proportional increase of lignin content in bitumen. The curves of modulus had a trend to move upward with the increase of lignin while those of the phase angle moved downward. It shows that the lignin could increase the modulus and elasticity of bitumen. Also, the aged samples had increased modulus and decreased phase angle, a phenomenon which is attributed to oxidative age hardening.

On the range of high frequencies, the properties between all samples were very close. On the contrary, the differences between all samples were larger in the low-frequency region. It shows that the effect of lignin on the high-temperature performance of bitumen is greater than that at low temperatures. Although the modulus increase improved the resistance to deformation, it may result to a propensity for brittle fracture at low temperatures. However, the effect of lignin on bitumen mainly reflected at high temperatures, showing improved high resistance to deformation. From the perspective of the entire frequency range, the properties of binders with different contents of organosolv lignin were not much different.

#### 3.2.2. Linear Amplitude Sweep

**Figure 5:**
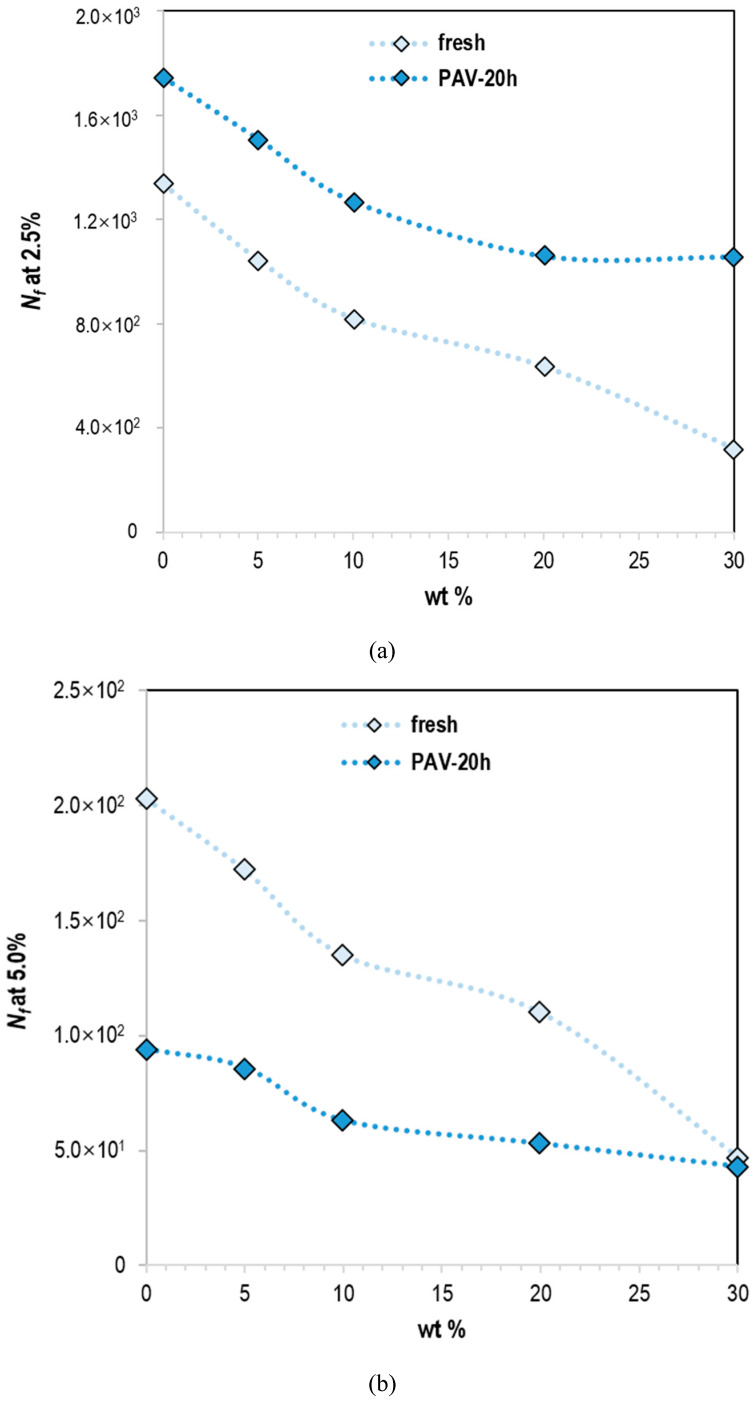
*N_f_* at (**a**) 2.5% and (**b**) 5.0% of the applied shear strain of lignin–bitumen systems before and after aging.

As mentioned earlier, the fatigue performance of lignin–bitumen systems was measured by linear amplitude sweep (LAS) tests. The number of cycles to failure (*N_f_*) of different samples at two different typical strain levels (2.5 & 5.0%) was illustrated in Figure 5. Obviously, the dramatic decrease of fatigue life was associated with the increase of strain level. It shows that the material is more likely to be destroyed at higher strain levels. It was also well understood that under real pavement conditions, vehicles with a heavier axle load were more likely to cause damage.

Fatigue life to failure decreased with the increase of lignin content at both high and low strain levels (2.5 & 5.0%). The addition of lignin led to stiffer binders, a performance confirmed by the master curves of modulus and phase angle mentioned earlier. At high temperatures, the stiffer material increased resistance to deformation. However, it may be prone to brittle fracture, which affects the medium-low temperature performance. From another perspective, lignin and bitumen were two different phase materials; one was a kind of powdery material, the other one was a kind of viscoelastic material. Even if the two materials were mixed uniformly and evenly, vulnerable interfaces still exist, making them more susceptible to damage under accumulated loads. With the same lignin content, the fatigue life increases at low strain levels and decreases at high strain levels, indicating that the stiffer binders were more vulnerable to fatigue failure at higher strain levels.

As shown in Figure 5 and Table 3, the fatigue life parameter A represents the original fatigue life at a strain level of 1%. For logarithmic coordinates, the strain sensitive factor B of the applied shear strain represents the slope. The slope indicates the material’s sensitivity to strain levels. The higher strain sensitivity factor B meant the faster and more the fatigue life decreased with the strain level increasing. By increasing the strain level, the fatigue life decreased further. The slope of the graph did not change significantly after aging, showing that the addition of lignin did not increase the sensitivity of the material to deformation.

Although the slope did not change dramatically, the lines moved downward and fatigue life reduced as the lignin content increases. Lignin did not affect the strain sensitivity but it resulted in a slight reduction of fatigue life. However, the aging process increased the sensitivity to the strain level, with the slope becoming steeper with the aging.

Different strain levels simulated vehicle loads with different axle loads. The heavier the axial load, the greater the strain level. For the same amount of lignin, the two lines in the fresh and aged conditions had an intersection point, which implied that the damage to fresh and aged binders can be the same under a specific strain level and axial load. At low strain levels, fresh binders were more susceptible to fatigue failure. However, at high strain levels, damage to aged materials was more severe. The material became stiffer and more brittle due to aging, and it appeared to increase the resistance to deformation at low strain levels, and the brittle fracture was more likely to occur at high strain levels. These intersections moved to higher strain levels as the lignin content increases.

#### 3.2.3. Multiple Stress Creep Recovery

**Figure 6:**
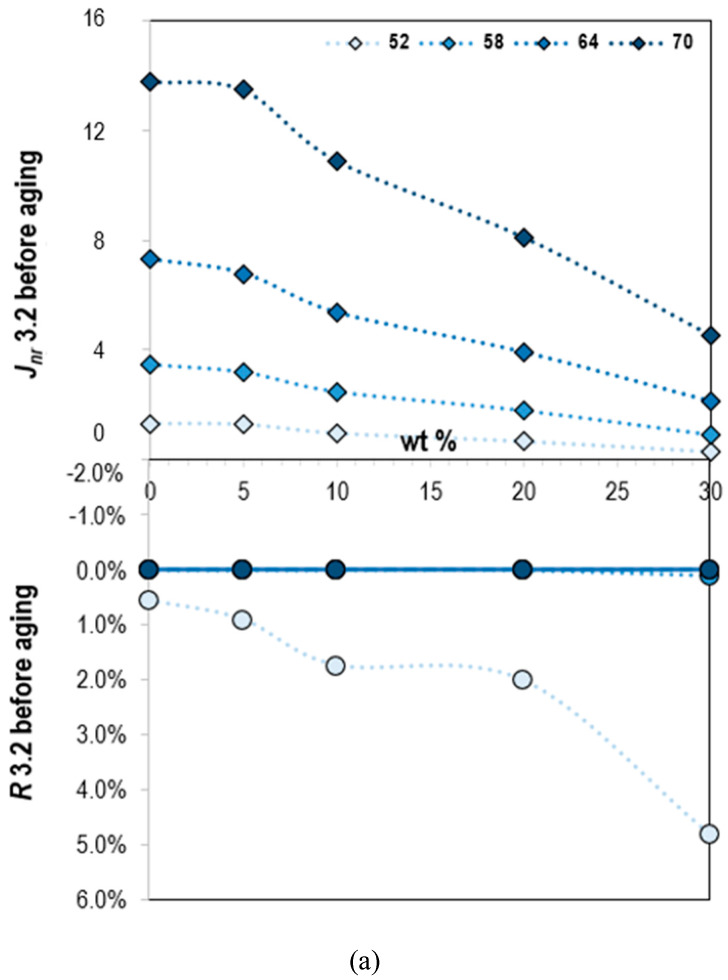

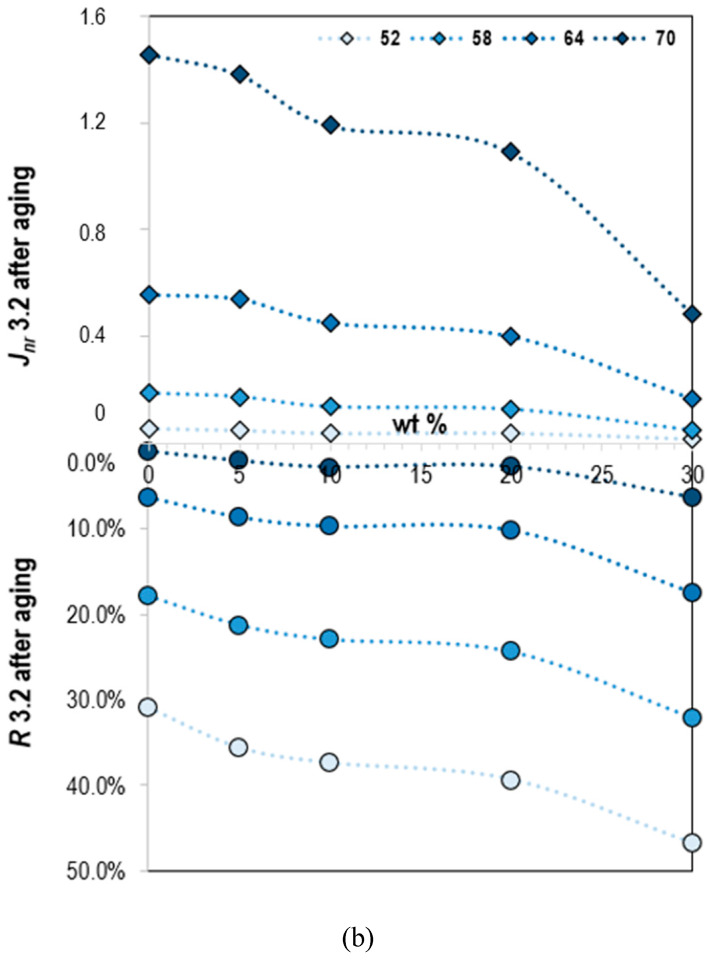
Nonrecoverable compliance (*J_nr_*) and recovery percentage (*R*) of lignin–bitumen systems (**a**) before and (**b**) after aging (stress level 3.2 kPa).

The recovery percentage (*R*) and nonrecoverable compliance (*J_nr_*), which indicate the recovery and nonrecovery performance, were measured by the multiple stress creep recovery (MSCR) test. The results of fresh and PAV aged samples at different stress levels (0.1 and 3.2 kPa) were plotted in Figure 6. 

A higher recovery percentage implied that the material showed more elasticity, while lower nonrecoverable compliance means that the material exhibited higher modulus. The results under different aging periods and stress levels have common characteristics and are slightly different from each other. Obviously, changes in temperature and stress levels had a significant impact on properties. Nonrecoverable compliance increased and recovery percentage decreased with increasing temperature. Bitumen is more viscous at high temperatures than at low temperature and thus more likely to deform. Moreover, the higher the stress level, the higher the nonrecoverable compliance, and the lower the recovery percentage. It shows that it is easier to cause unrecoverable plastic deformation of the material at high stress levels. The nonrecoverable compliance of lignin–bitumen systems decreased and the recovery percentage increased due to oxidative age hardening and the impact of oxidation was apparent.

The influence of lignin content was analyzed as well, and the results revealed that it has a marked effect on the recovery and nonrecovery ability of the bitumen. Under the same external test conditions, the recovery percentage and the nonrecoverable compliance increased and decreased, respectively with the increase of lignin content. The addition of lignin makes the material stiffer and the recoverability significantly enhanced, which can be confirmed from the master curves as well. It is concluded that the lignin increases the recoverability of the binders and improves the rutting resistance at high temperatures.

## 4. Conclusions

The retardation of oxidative aging of bitumen was found to be enhanced by the addition of a relatively small amount of an organosolv lignin. Based on spectroscopic measurements, the addition of lignin in bitumen reduced the production rate of carbonyl and sulfoxide compounds and it had a certain oxidation inhibition effect on the bitumen. To quantify the effect of lignin on bitumen, the *IRI* of aged functional groups was calculated to evaluate the actual effect of lignin as an oxidation inhibitor, and it was concluded that the dosage of lignin was 10% by the mass of bitumen, showing the lowest *IRI* of aged functional groups. Although the exact lignin-induced inhibition mechanism in bitumen remains unknown, lignin definitely suppresses the oxidation of carbon and sulfur species in bitumen, most possibly by interacting physically with these species which, otherwise, would be readily oxidizable. 

The rheological characteristics of lignin–bitumen samples both at low and high temperatures were evaluated as well proving that lignin did not degrade the mechanical response of bitumen before and after aging. According to the frequency sweep results presented above, the organosolv lignin could increase the modulus and decrease the phase angle at a wide frequency range. Regarding the response of binders at low and medium temperatures, a slight reduction of fatigue life of bitumen was noticed, something which potentially results to increased sensitivity to brittle fracture. Nevertheless, lignin had a significant effect on the recovery and nonrecovery ability of the bitumen. Lignin increased the recoverability and hardness of the lignin binders and improved the rutting resistance at high temperatures.

Overall, only one kind of lignin was used in this study. Other types of lignin extracted by different methods, such as kraft, soda, and lignosulfonate lignin, will be tested in future research. The miscibility between lignin and bitumen will be researched further, including the storage modulus and separation after homogenously mixing. While bituminous binders with different properties, the effect of lignin on soft and hard binders can be assessed in the future as well. 

## Figures and Tables

**Table 1:** Sample tags.

Lignin by Bitumen Mass [%]	Tag
0	Bref
5	BL05
10	BL10
20	BL20
30	BL30

**Table 2:** Main functional groups of bitumen in Fourier transform infrared (FTIR) spectra.

Wavenumber (cm^−1^)	Functional Groups
2990–2880	Stretching aromatic
2880–2820	Stretching symmetric
1753–1660	Oxygenated functional group (carbonyl)
1670–1535	Aromatic structures
1525–1395	Aliphatic structures
1390–1350	Branched aliphatic structures
1047–995	Oxygenated functional group (sulfoxide)
912–838	Out of singlet
838–783	Out of adjacent
783–734	Out of adjacent
734–710	Long chains

**Table 3:** Fatigue fitting functions of studied materials.

Studied Materials	N*_f_* = *A*(*γ*)^−B^	
	Before Aging	After Aging
Bref	N*_f_* = 16132(*γ*)^−2.7194^	N*_f_* = 83264(*γ*)^−4.2192^
BL05	N*_f_* = 11317(*γ*)^−2.5995^	N*_f_* = 66378(*γ*)^−4.1306^
BL10	N*_f_* = 8946(*γ*)^−2.6073^	N*_f_* = 67739(*γ*)^−4.3408^
BL20	N*_f_* = 6437(*γ*)^−2.5262^	N*_f_* = 55829(*γ*)^−4.3258^
BL30	N*_f_* = 4071(*γ*)^−2.7727^	N*_f_* = 73869(*γ*)^−4.6345^

## References

[1] Zhang Y., Liu X., Apostolidis P., Gard W., Van De Ven M., Erkens S., Jing R. (2019). Chemical and Rheological Evaluation of Aged Lignin-Modified Bitumen. Materials.

[2] Xu G., Wang H., Zhu H. (2017). Rheological Properties and Anti-Aging Performance of Asphalt Binder Modified with Wood Lignin. Constr. Build. Mater..

[3] Van Vliet D., Slaghek T., Giezen C., Haaksman I. Lignin as a Green Alternative for Bitumen. Proceedings of the 6th Euroasphalt & Eurobitume Congress.

[4] Bruijnincx P., Weckhuysen B., Gruter G.J., Engelen-Smeets E. (2016). Lignin Valorisation: The Importance of a Full Value Chain Approach.

[5] Apostolidis P., Liu X., Kasbergen C., Scarpas A.T. (2017). Synthesis of Asphalt Binder Aging and the State of the Art of Antiaging Technologies. Transp. Res. Rec..

[6] Sundstrom D.W., Klei H.E., Daubenspeck T.H. (1983). Use of Byproduct Lignins as Extenders in Asphalt. Ind. Eng. Chem. Prod. Res. Dev..

[7] Williams R.C., McCready N.S. (2008). The Utilization of Agriculturally Derived Lignin as an Antioxidant in Asphalt Binder. Trans. Proj. Rep..

[8] Gosselink R. (2011). Lignin as a Renewable Aromatic Re-Source for the Chemical Industry. Ph.D. Thesis.

[9] Batista K.B., Padilha R.P.L., Castro T.O., Silva C., Araújo M., Leite L., Pasa V., Lins V. (2018). High-Temperature, Low-Temperature and Weathering Aging Performance of Lignin Modified Asphalt Binders. Ind. Crop. Prod..

[10] Boeriu C.G., Bravo D., Gosselink R.J.A., van Dam J.E.G. (2004). Characterization of Structure-Dependent Functional Properties of Lignin-Natural Antioxidants. Ind. Crop. Prod..

[11] Dizhbite T., Telysheva G., Jurkjane V., Viesturs U. (2004). Characterization of the Radical Scavenging Activity of Lignins-Natural Antioxidants. Bioresour. Technol..

[12] Pan T. (2012). A First-Principles based Chemophysical Environment for Studying Lignins as an Asphalt Antioxidant. Constr. Build. Mater..

[13] Peterson J.C. (2009). A Review of the Fundamentals of Asphalt Oxidation: Chemical, Physicochemical Property, and Durability Relationship. Transportation Research Circular E-C140.

[14] Kun D., Pukánszky B. (2017). Polymer/Lignin Blends: Interactions, Properties, Applications. Eur. Polym. J..

[15] Mandlekar N., Cayla A., Rault F., Giraud S., SalaüN F., Malucelli G., Guan J.P. (2018). An Overview on the Use of Lignin and its Derivatives in Fire Retardant Polymer Systems. Lignin-Trends Appl..

[16] Sheldon R.A. (2018). The Road to Biorenewables: Carbohydrates to Commodity Chemicals. ACS Sustain. Chem. Eng..

[17] ASTM International (2019). Standard Practice for Accelerated Aging of Asphalt Binder Using a Pressurized Aging Vessel (PAV): ASTM D 6521-19.

[18] AASHTO (2019). Standard Method of Test. for Determining the Rheological Properties of Asphalt Binder Using a Dynamic Shear Rheometer (DSR): AASHTO T 315-19.

[19] AASHTO (2014). Standard Method of Test. for Estimating Damage Tolerance of Asphalt Binders Using the Linear Amplitude Sweep: AASHTO TP 101-14.

[20] ASTM International (2015). Standard Test. Method for Multiple Stress Creep and Recovery (MSCR) of Asphalt Binder Using a Dynamic Shear Rheometer: ASTM D 7405-15.

